# Safety and efficacy of intravesical instillation of resiniferatoxin in healthy cats: A preliminary study

**DOI:** 10.3389/fvets.2022.922305

**Published:** 2023-01-12

**Authors:** Michele Barletta, Julie Gordon, André Escobar, Krista Mitchell, H. Nicole Trenholme, Janet A. Grimes, Juan M. Jiménez-Andrade, Alexis Nahama, Alvaro Cisternas

**Affiliations:** ^1^Department of Large Animal Medicine, College of Veterinary Medicine, University of Georgia, Athens, GA, United States; ^2^Department Small Animal Medicine and Surgery, College of Veterinary Medicine, University of Georgia, Athens, GA, United States; ^3^Unidad Académica Multidisciplinaria Reynosa-Aztlán, Universidad Autónoma de Tamaulipas, Reynosa, Tamaulipas, Mexico; ^4^ARK Animal Health, Sorrento Therapeutics, San Diego, CA, United States

**Keywords:** C-fibers, feline, interstitial cystitis, pain, substance P, urinary bladder, calcitonin gene-related peptide (CGRP)

## Abstract

**Objectives:**

To evaluate the safety of intravesical application of resiniferatoxin (RTX) in healthy cats and its effects on calcitonin gene-related peptide (CGRP) and substance P (SP) produced by C-fibers.

**Methods:**

Seven adult female cats received either 25 mL of saline (control; *n* = 1), or intravesical RTX at 5, 25, or 50 μg in 25 mL of saline to a final concentration of 0.2 μg/mL (318 nM), 1 μg/mL (1,591 nM), and 2 μg/mL (3,181 nM) (*n* = 2 per group). The treatment was instilled into the urinary bladder for 20 min. Plasma concentrations of RTX were measured at 0, 0.5, 1, and 4 h. Physical exam, complete blood count, and serum biochemical analysis were performed on day 0, 7, and 14. After 14 days, the sacral dorsal root ganglia (DRG) and the urinary bladder were harvested for histological and immunofluorescence analysis.

**Results:**

Intravesical RTX was well tolerated and plasma concentrations were below the quantifiable limits except for one cat receiving 1 μg/mL. Mild to moderate histopathological changes, including epithelial changes, edema, and blood vessel proliferation, were observed at lower doses (0.2 and 1 μg/mL), and were more severe at the higher dose (2 μg/mL). C-fiber ablation was observed in the urinary bladder tissue at all doses, as shown by an apparent reduction of both CGRP and SP immunoreactive axons.

**Conclusion:**

A dose of 25 μg (1 μg/mL) of RTX instilled in the urinary bladder of healthy cats appeared to decrease the density of SP and CGRP nerve axons innervating bladder and induced moderate changes in the bladder tissue.

## Introduction

Resiniferatoxin (RTX) is a natural occurring diterpene isolated from the latex of different species of Euphorbia, a cactus-like plant found in Morocco. Its structure is related to the phorbol esters, with esterification at the 20-hydroxyl group position with a 4-hydroxy-3-methoxyphenylacetate, which makes this molecule an irritant (1). RTX is a potent agonist of transient receptor potential vanilloid 1 (TRPV1), a calcium-permeable non-selective cation channel. These receptors are located on primary afferent neurons responsible for nociception (2). Cytotoxicity of TRPV1-expressing neurons is caused by the prolonged calcium-influx induced by RTX binding (3), and the destruction of myelinated Aδ-fibers and unmyelinated C-fibers expressing these receptors has shown to block clinically relevant pain while sparing somatic sensitization (4–6). The efficacy of RTX has been reported for both central, *via* intrathecal injection (5, 7, 8), and peripheral route. The latter use decreases systemic adverse effects and allows for treatment of localized pain. Peripheral injection of RTX has shown strong efficacy for surgical pain (9), thermal injury (10), and arthritis (11) in rodents and long term pain relief in dogs with osteoarthritis (12).

Substance P (SP), a neuropeptide that mediates nociception and neurogenic inflammation, co-localizes with TRPV1-expressing primary afferent neurons (13). Loss of SP immunoreactivity is therefore considered to be an indication of a loss of small TRPV1(+) peptidergic neurons (14–17). Similarly, the calcitonin gene-related peptide (CGRP) is localized in C-type sensory neurons (15, 16). Previous studies in mice have also reported that the great majority of CGRP-immunoreactive axons in myenteric ganglia are also TRPV1-immunoreactive (18).

Feline interstitial cystitis (IC) is a multifactorial syndrome where the neuroaxis is sensitized to sensory inputs (19). The exact etiology is unknown; however, intrinsic and extrinsic abnormalities, and environmental changes may play a role (19). Cats affected by this syndrome have an increase in high affinity SP binding sites in the urothelium (20, 21).

It has been shown that the sacral region, primarily S2, provides the main source of afferent innervation to the urinary bladder and urethra in the cat (22). Furthermore, in subjects affected with IC, cell bodies in the dorsal root ganglia (DRG) between L4 and S3 had an altered neuropeptide profile and were 28% larger than those from normal cats (23).

The goals of the current study were to assess the safety of RTX instilled into the urinary bladder, as well as the effects on SP and CGRP immunoreactivity in cell bodies and their axons innervating bladder in healthy female cats. The specific aims of the study were: (1) to describe the clinical tolerability, (2) to measure plasma concentrations, (3) to visualize and quantify axons and cell bodies expressing SP and CGRP in the urinary bladder and sacral DRG, and (4) to assess the cellular integrity of the urinary bladder tissue after intravesical application of RTX. We hypothesized that RTX would be well tolerated with minimal systemic absorption, a decrease in SP and CGRP would occur in both the urinary bladder and corresponding DRG, and minimal to no histological changes would be noted in the urinary bladder tissues after instillation of RTX.

## Materials and methods

### Animals

A pilot study was conducted to determine the location and presence of SP and CGRP in the normal feline urinary bladder and sacral DRG (S1 and S2). Two healthy adult sexually intact female cats were euthanized *via* total body perfusion under general anesthesia using 1 L of NaCl 0.9% followed by 1 L of 10% neutral buffered formalin. At the end of the procedure, the urinary bladder, S1, and S2 were harvested and submitted for immunofluorescence analysis.

Seven adult sexually intact female cats, weighing 3.1 ± 0.17 kg, between 2 and 5 years of age, were enrolled in the study. Animals were deemed healthy based on physical examination and blood testing including a complete blood count and serum biochemical analysis. Cats were housed in groups of two or three subjects per cage and were acclimated for a minimum of 7 days in an animal housing facility at the University of Georgia, College of Veterinary Medicine. A commercially available maintenance diet was offered twice a day and water provided *ad libitum*.

### Study design

A prospective, masked, randomized design was used for this study. Anesthesia was induced with 5 mg/kg of ketamine, 0.3 mg/kg of butorphanol, and 7 μg/kg of dexmedetomidine IM. After orotracheal intubation, general anesthesia was maintained with isoflurane in 100% oxygen delivered *via* a rebreathing system. Intravenous catheters were placed in the cephalic vein and in the jugular vein for administration of lactated Ringer's solution at a rate of 3 mL/kg/h and blood collection for RTX plasma analysis, respectively. End tidal carbon dioxide, heart rate, respiratory rates, SpO_2_, ECG, and non-invasive blood pressure were recorded throughout the procedure using a multiparameter monitor (SurgiVet Advisor vital signs monitor, Smiths Medical, Saint Paul, MN, USA). Animals were assigned to receive an intravesical instillation of RTX (Resiniferatoxin, ARK Animal Health, San Diego, CA, USA) (6 subjects) or an equivalent volume of sterile saline (control, 1 subject). The RTX used in the current study was formulated in accordance with the Good Manufacturing Practice (GMP) regulations and contained 3% Polysorbate 80. Cats receiving RTX were divided into three treatment groups (two subjects per group): 5, 25, and 50 μg of RTX total. The study drug was stored in a dedicated refrigerator and maintained at all time at a temperature between 2 and 8°C (average temperature 4°C). RTX was mixed with 25 mL of saline immediately prior to administration to a final concentration of 0.2 μg/mL (318 nM), 1 μg/mL (1,591 nM), and 2 μg/mL (3,181 nM), respectively.

After preparing the area using chlorhexidine solution, a 4 or 5 French Foley urinary catheter (Foley Catheter with wire stylet, Mila International, Inc., Florence, KY, USA) was placed using aseptic technique. The catheter balloon was inflated with the manufacture's recommended volume of saline and the urinary bladder was emptied. Before the RTX administration, 25 mL of sterile saline were instilled in the urinary bladder over 60 s and then removed. After flushing the bladder, the treatment was instilled over 60 s and left in the urinary bladder for 20 min. The bladder was then emptied and the subject was kept under general anesthesia for an additional 60 min. At the end of this period, the indwelling catheter was removed and the subject was allowed to recover and returned to their cage.

Cats were observed twice daily for 14 days and were monitored for decrease in food consumption and defecation, urinary incontinence, stranguria, and changes in behavior, such as decrease in activity and grooming. During this assessment, the abdomen was palpated for signs of discomfort, including vocalization and aggressive behavior. Blood samples were collected at day 7 and 14 for complete blood count and serum biochemical analysis.

### Drug analysis

A baseline blood sample was collected from the jugular catheter before treatment administration. Blood was also collected at 30 min, 1 h, and 4 h after intravesical instillation of RTX. A 1 mL sample was discarded prior to collection of 2 mL, which was immediately transferred to blood tubes (BD Vacutainer, 2.0 mL, Becton Dickinson and Co., Franklin Lakes, NJ, USA) containing K_2_EDTA. Samples were centrifuged for 10 min at 1,500 g within 5 min after collection. The plasma obtained was homogenized *via* pipetting, aliquoted into 1.5 mL cryogenic vials, and frozen at −80°C.

Plasma samples containing RTX were spiked with RTX-d_5_ as internal standard and were processed using a supported liquid extraction procedure using a 96-well plate (Novum ™ SLE Mini 96-well plate, Phenomenex, Torrance, CA, USA). A mixture of hexane:MTBE (650:350, v/v) was used to extract the plasma sample, which was then dried under nitrogen. Extracts were analyzed by high performance liquid chromatography using a Waters CORTECS UPLC C18 column. The mobile phase was nebulized using heated nitrogen in electrospray positive ionization mode and compounds were detected using tandem mass spectrometry (Waters Xevo TQ system, Waters Corporation, Milford, MA, USA). Samples were assayed in triplicate and the average was reported as the sample concentration. The peak areas of RTX and the internal standard (IS) were acquired using commercially available software (Analyst v. 1.6.2, AB Sciex, Framingham, MA, USA). The calibration curves were obtained by fitting the peak area ratios of RTX/IS and the standard concentrations to a linear weighted (1/ × 2) equation using software (Analyst).

The correlation coefficient of the calibration curve was 0.999. The back-calculated calibration standard concentrations were within ± 8% of the actual values and indicated that the RTX standard and response could be described by a linear weighted (1/ × 2) equation for concentrations ranging from 50.0 to 10,000 pg/mL (0.08–16 nM). The accuracy of the method was determined by comparing the mean measured concentrations with the theoretical concentrations of the compound in the quality control (QC) samples. The theoretical concentrations used were 150, 800, and 8,000 pg/mL (0.24, 1.27, and 12.72 nM) and the measured concentrations were reported as the average of three replicates. For RTX QC samples, the deviation of the mean from theoretical values did not exceed 4.00%. For the QC samples prepared in feline plasma, the precision of the method was determined from the percent coefficient of variation (%CV) of the QC sample replicates at each concentration level. The %CV for RTX QC samples ranged from 0.462 to 2.50%. The limit of quantification for this method was 50 pg/mL (0.08 nM).

### Tissue collection and preparation

Euthanasia was performed 14 days after RTX administration *via* total body perfusion with the animal under general anesthesia. The same anesthetic protocol described for the instillation of RTX in the urinary bladder was used for this procedure. A lateral thoracotomy at the level of the 4th intercostal space was performed, a cannula was inserted into the aorta and secured with umbilical tape. The blood was cleared by administration through the cannula of 1 L of NaCl 0.9% between 18.7 and 21.5 kPa (140 and 160 mmHg) of pressure followed by 1 L of 10% neutral buffered formalin. At the same time, an incision was made into the right ventricle to drain the blood and excess sterile saline. After confirmation of successful euthanasia, the sacral DRG S1 and S2 and the body, trigone, and fundus of the urinary bladder were harvested and submitted for immunofluorescence and histological analysis.

Tissue samples were placed into separate containers and fixed in 10% formalin at 4°C for 24 h. The following day, samples were transferred to 0.1 M PBS and stored at 4°C for up to 3 days. Tissues were then washed in fresh 0.01 M PBS and immediately cryopreserved in a 40% sucrose solution for at least 2 days and no more than 7 days at 4°C. Bladders were then cut into serial cross sections (30 μm thick) with a cryostat (Leica cryostat, CM1900, Wetzlar, Germany) in an anatomical frontal plane. S1 and S2 DRG were cut into serial cross sections (15 μm thick) in an anatomical sagittal plane.

### Histological analysis

Tissue sections from the body, fundus, and trigone of the urinary bladder were stained with hematoxylin and eosin. A semiquantitative histopathological analysis was performed by a medical pathologist with more than 30 years of experience who was masked to the treatment groups. Briefly, mild dysplasia of the basal membrane and epithelium of the cat urinary bladder was defined when the cell nuclei were hyperchromatic and enlarged (Tables 1, 2). Furthermore, fibrosis in the lamina propria was graded based on the number of fibroblasts with an irregular shape (elongated) per visual field; mild (<5 cells), moderate (between 5 and 10 cells) and severe (more than 10 cells). Edema of muscle layers and blood vessels were subjectively graded based on the separation space between muscle fascicles and the amount of fluid surrounding the vessels, respectively. Finally, proliferation of blood vessels was defined as mild (<5 blood vessels), moderate (between 5 and 10 blood vessels) and severe (more than 10 blood vessels per field of view).

**Table 1:** Histopathological analysis of the basal membrane and epithelium of the bladder of cats 14 days after treatment with 0 (control), 0.2, 1, and 2 μg/mL of RTX instilled into the urinary bladder.

**Subject (treatment)**	**Tissue**	**Integrity of basal membrane**	**Integrity of epithelial tissue**	**Dysplasia**
3 (control)	Body	No alterations	Intact	–
	Fundus	No alterations	Intact	–
	Trigone	No alterations	Absence of epithelium	–
2 (0.2 μg/mL)	Body	No alterations	Loss of cohesion	–
	Fundus	No alterations	Epithelial thinning	–
	Trigone	No alterations	Intraepithelial edema	–
5 (0.2 μg/mL)	Body	No alterations	Epithelial thinning	–
	Fundus	No alterations	Absence of epithelium	–
	Trigone	No alterations	Epithelial thinning	–
1 (1 μg/mL)	Body	Partial rupture	Massive detachment of epithelium	–
	Fundus	No alterations	Focal detachment of epithelium	–
	Trigone	No alterations	Loss of cohesion	+
7 (1 μg/mL)	Body	No alterations	Loss of cohesion	+
	Fundus	No alterations	Detachment of epithelial cells	+
	Trigone	No alterations	Epithelial thinning	–
4 (2 μg/mL)	Body	Total loss	Massive detachment of epithelium	+
	Fundus	No alterations	Poor hyperplasia of epithelium	+
	Trigone	No alterations	Moderate hyperplasia of epithelium	+
6 (2 μg/mL)	Body	No alterations	Massive detachment of epithelium	–
	Fundus	No alterations	Absence of epithelium	–
	Trigone	No alterations	Massive detachment of epithelium	+

Tissue sections were stained with hematoxylin and eosin. Histological analysis was performed by a medical pathologist who was masked to the treatment groups.

–Absent; + mild.

**Table 2:** Histopathological analysis of the lamina propria, muscular layers, and blood vessels of the urinary bladder of cats 14 days after treatment with 0 (control), 0.2, 1, and 2 μg/mL of RTX instilled into the urinary bladder.

**Subject (treatment)**	**Tissue**	**Fibrosis of lamina propria**	**Edema of lamina propria**	**Mucus formation**	**Edema of muscle layers**	**Edema of blood vessels**	**Proliferation of blood vessels**
3 (control)	Body	–	+	–	–	–	–
	Fundus	–	–	–	–	–	–
	Trigone	–	–	–	–	–	+
2 (0.2 μg/mL)	Body	–	+	+	–	–	+
	Fundus	–	–	–	–	+	–
	Trigone	–	–	–	–	+	–
5 (0.2 μg/mL)	Body	–	–	–	–	+	–
	Fundus	–	–	–	+	+	–
	Trigone	–	–	–	+	+	–
1 (1 μg/mL)	Body	++	–	–	+	–	+
	Fundus	+	–	–	+	–	+
	Trigone	–	–	+	+	–	+
7 (1 μg/mL)	Body	–	–	–	+	–	+
	Fundus	–	–	–	+	–	+
	Trigone	–	+	–	+	–	+
4 (2 μg/mL)	Body	+	–	+++	+	–	+
	Fundus	+	–	+	+	–	+++
	Trigone	–	–	++	+	–	–
6 (2 μg/mL)	Body	–	+++	+	++	–	–
	Fundus	+	+	–	+	–	–
	Trigone	++	–	+	+	–	+

Tissue sections were stained with hematoxylin and eosin. Histological analysis was performed by a medical pathologist who was masked to the treatment groups.

–Absent; + mild; ++ moderate; +++ severe.

### Immunofluorescence quantification

Both SP (rabbit-anti-rat SP, catalog # 20064, ImmunoStar, Hudson, WI, USA) (24) and CGRP (rabbit-anti-rat CGRP, catalog # C8198, Sigma Aldrich, St. Louis, MO, USA) (25) antibodies were used to indirectly identify TRPV1 axons innervating the feline urinary bladder and TRPV1 positive cell bodies in the S2 DRG. SP and CGRP immunoreactivity of bladder and DRG tissue was visualized in cross-sectional frozen sections using indirect immunofluorescence. Sections were mounted onto glass slides, incubated with 5% blocking solution (5% normal donkey serum, 0.5% Triton X100 in 0.1 M PBS) for 2 h, and then incubated with appropriate primary antibodies (SP 1:3,000 or CGRP 1:3,000) for 12 h. After this period, sections were incubated with a Cy3 monoclonal donkey anti-rabbit secondary antibody (Cy3 AffiniPure donkey anti-rabbit IgG H+L catalog # 711-165-152, Jackson ImmunoResearch Laboratory, Inc., West Grove, PA, USA) (1:600). Slides were then dehydrated through an alcohol gradient (70, 80, 90, and 100% ethanol), rinsed in xylene, and cover slipped using dibutylphthalate polystyrene xylene mounting medium.

Images were obtained for each marker using a scanning confocal laser microscope (Carl Zeiss scanning confocal laser microscope, model LSM 800, Jena, Germany) with the Z stack function. To quantify the density of SP and CGRP-immunoreactive axons in the different regions of feline urinary bladder, four samples per subject were initially observed at low magnification (10×) to allow identification of areas with the greatest density of nerve fibers. When the target area was identified, an image was obtained for each section at 40× magnification and analyzed using commercially available software (ImageJ software, U.S. National Institutes of Health, Bethesda, MD, USA). Each nerve fiber was traced manually using the freehand line tool to determine its length. In each image, the area of interest was determined by using the built-in area tool, and then multiplied by the thickness of the section (30 μm) that was kept constant to obtain the volume. The presence of the nerve fibers in the calculated volume was expressed as density and it was reported as the total length of the nerve fibers by volume of urinary bladder analyzed (mm/mm^3^). Finally, the average of the four sections analyzed per subject (for each given marker) was obtained. All quantifications were done masked to the treatment condition.

To quantify the expression of SP and CGRP at S1 and S2 DRG, the number of SP and CGRP positive cell bodies in this tissue was manually counted in serial 15 μm sections from a minimum of four sections per animal using a fluorescence microscope. The quantification was performed by an experienced technician and only cells with a circular shape, having a diameter between 15 and 60 μm, and with a positive 4′,6-diamidino-2-phenylindole nucleus were included in the analysis. Non-neuronal cells, such as endothelial cells in the blood vessels, macrophages, and satellite glial cells, which have a different morphology and size as compared to cell bodies of the DRG neurons, were excluded. The number of SP or CGRP positive cell bodies was expressed as a percentage of the total number of DRG cell bodies.

### Data analysis

Individual data are presented for each animal (GraphPad Prism version 8.0; GraphPad Software, Inc., CA, USA). Due to the small number of animals in each group, statistical analysis was not performed.

## Results

General anesthesia was successfully induced in all subjects without complications. The first subject undergoing the procedure received 1 μg/mL (1,591 nM) of RTX and was maintained on a light plane of anesthesia to avoid hypotension. After RTX instillation, the heart rate increased from 110 beats/min to 230 beats/min and blood pressure initially decreased to a mean between 4 and 5.3 kPa (30–40 mmHg) for 15 min and then became non-detectable. This cardiovascular response persisted for ~45 min before returning to normal values and back to baseline values ~65 min after administration. The subject recovered from anesthesia without obvious complications. All subsequent animals were maintained on a deeper plane of anesthesia and the cardiovascular response observed in the first subject was not noted in any of the other cats. In addition, all subsequent subjects received an intramuscular injection of 3 mg/kg of ketamine ~20 min prior to RTX administration to prevent a possible nociceptive response. No other complications were noted for any of the other subjects during the procedure or in recovery.

No clinical signs of urinary discomfort, such as stranguria, pollakiuria, or urinary incontinence were noted during the 14-day follow up period. None of the subjects showed pain upon abdominal palpation, such as avoidance, vocalization, or aggressive behavior. Normal amount of urine without any traces of blood was observed daily in the litter boxes. Food and water consumption remained normal during this time. Complete blood count and serum biochemical analysis were within normal limits for all subjects at all timepoints.

Plasma concentrations following intravesical application of RTX were below the quantifiable limits for all subjects and at all timepoints except for one cat receiving 1 μg/mL (1,591 nM). In this subject, RTX plasma concentrations reached 55.5 pg/mL (0.09 nM) at 30 min after treatment. No RTX was detected in any of the plasma samples analyzed at 4 h after RTX instillation in the urinary bladder.

Intravesical instillation of RTX at the dose of 0.2 μg/mL (318 nM) resulted in minor change to the epithelial tissue and minor edema of blood vessels in the bladder. At the dose of 1 μg/mL (1,591 nM), RTX produced moderate change to the epithelial tissue, mild fibrosis of the lamina propria, and edema and proliferation of blood vessels. At the dose of 2 μg/mL (3,181 nM), RTX produced severe change to the epithelial tissue, severe edema and fibrosis of lamina propria, severe mucus formation, edema of muscle layers, as well as severe proliferation of blood vessels (Tables 1, 2).

**Figure 1:**
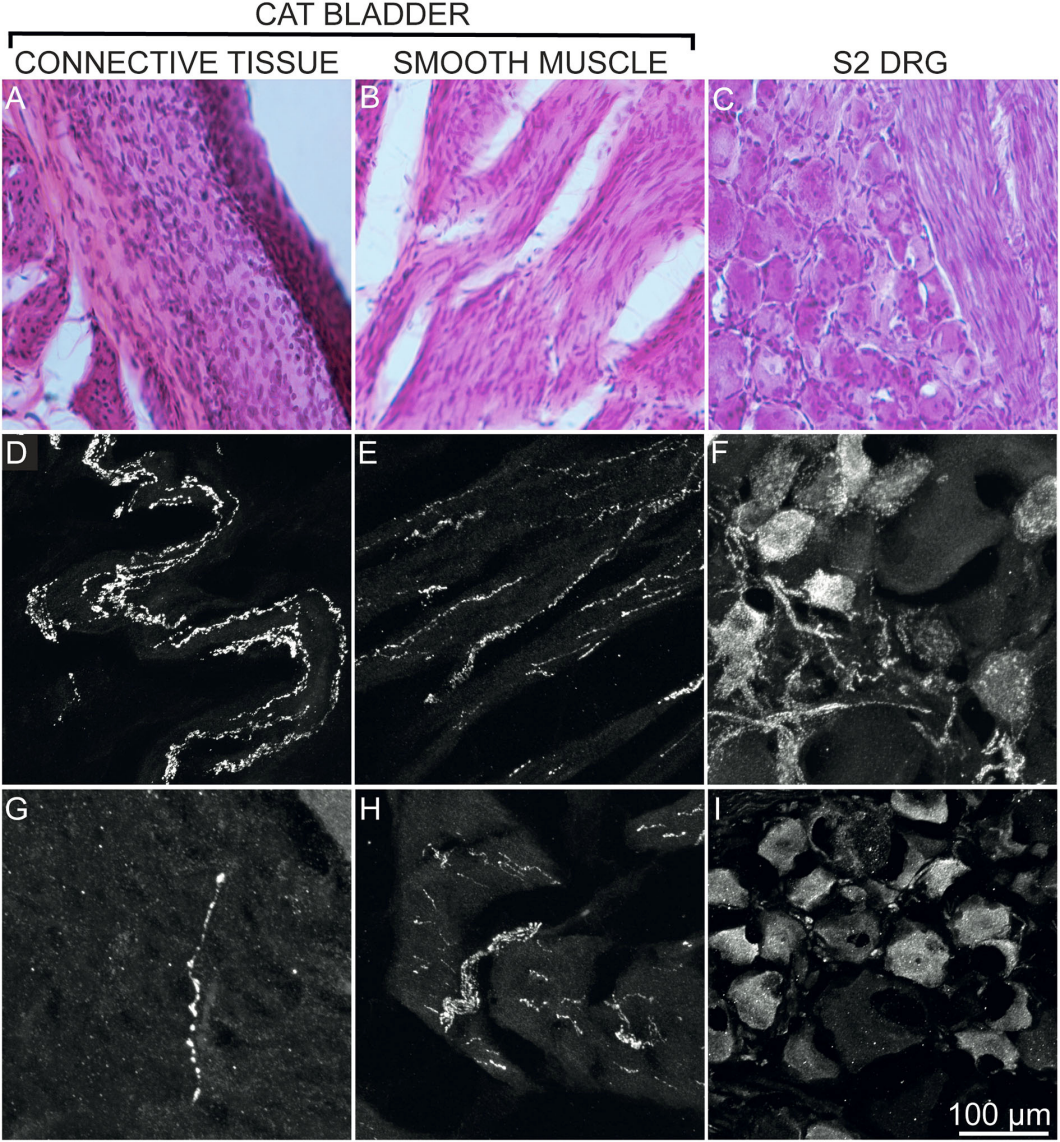
Expression of CGRP and SP by axons innervating the cat bladder (connective tissue **A, D, G**; smooth muscle **B, E, H**) and by cell bodies of S2 DRG **(C, F, I)** of 2 healthy adult sexually intact female cats (pilot study). Images of hematoxylin and eosin-stained sections are shown to illustrate the regions used for confocal images **(A–C)**. Confocal images revealed an apparent more abundant presence of CGRP immunoreactive axons **(D, E)** and immunoreactive cell bodies **(F)** as compared to SP expression **(G–I)**. Confocal images were obtained at 40× magnification using a Carl Zeiss scanning confocal laser microscope with Z-stack function. All panels represent tissues harvested from a healthy untreated cat (pilot subject).

Immunofluorescence analysis of the specimens obtained from the two pilot subjects revealed robust expression of CGRP and SP-immunoreactive axons innervating both connective tissue and smooth muscle of feline urinary bladder. Robust expression of these markers was also detected in the cell bodies of the sacral DRG (Figure 1).

**Figure 2:**
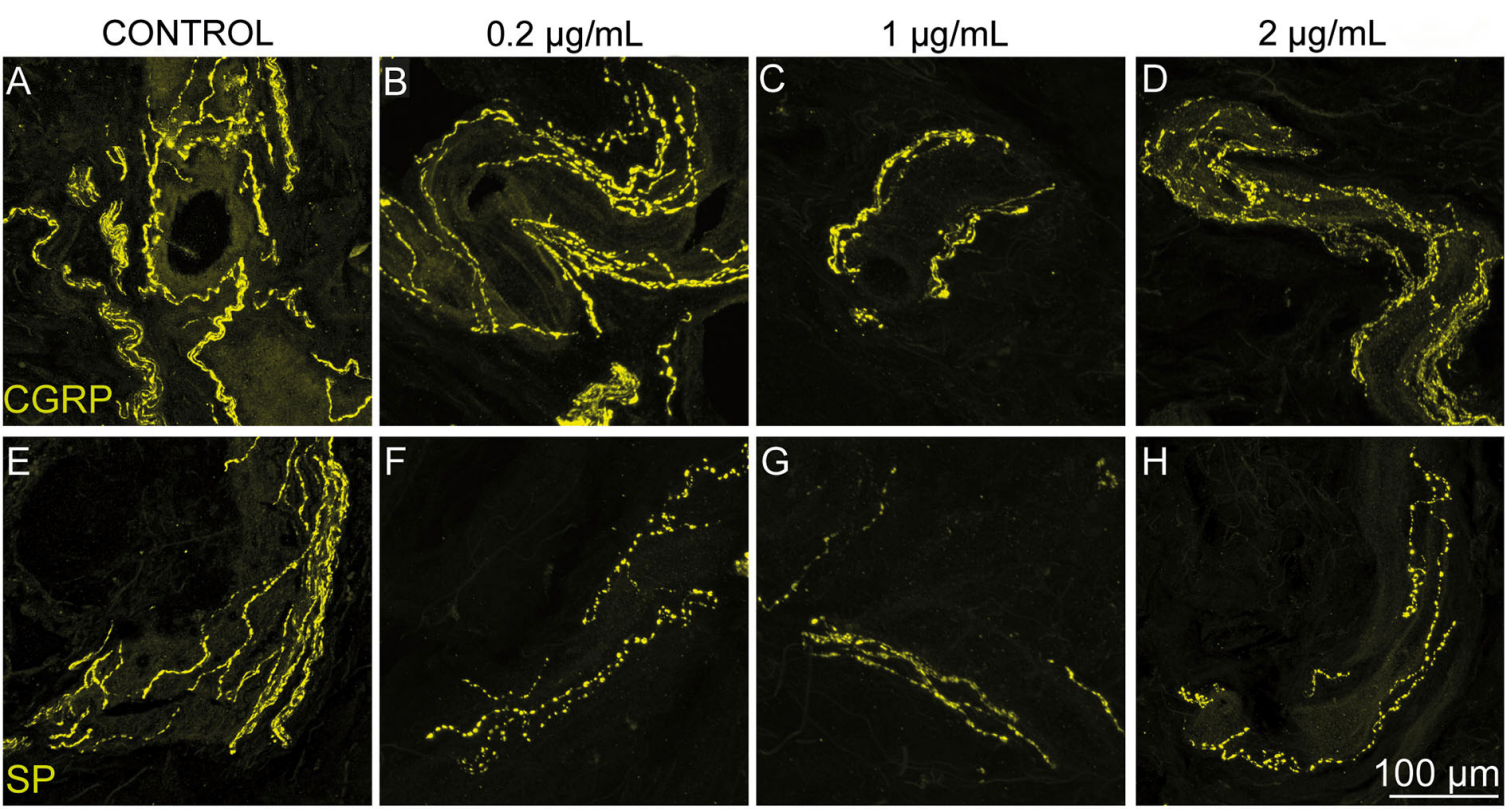
Intravesical RTX treatment appeared to decrease the density of both CGRP- and SP-immunoreactive axons innervating the body of the urinary bladder of healthy adult sexually intact female cats. Representative confocal images showing CGRP **(B–D)** and SP **(F–H)** axons innervating the bladder body after administration of saline (control **A, E**; *n* = 1) 0.2 (**B, F**; *n* = 2), 1 (**C, G**; *n* = 2), and 2 (**D, H**; *n* = 2) μg/mL of RTX. An apparent reduction in the density of both markers was seen in cats treated with RTX (14 days post-treatment) when compared to the control (saline) animal **(A, E)**. Confocal images were obtained at 40× magnification using a Carl Zeiss scanning confocal laser microscope with Z-stack function.

**Figure 3:**
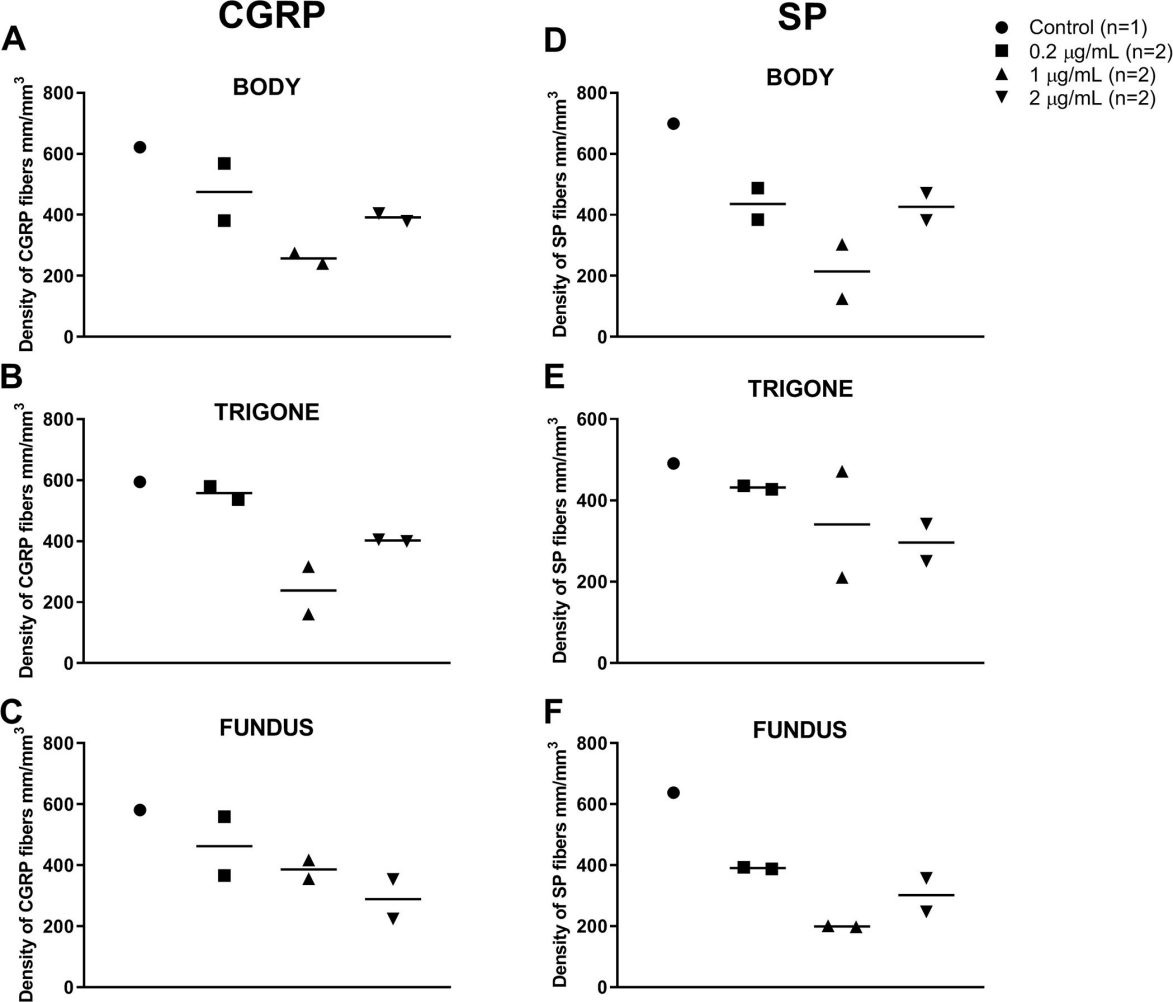
Individual values and median of the density of nerve fibers in the urinary bladder of 7 healthy adult sexually intact female cats expressing CGRP **(A–C)** and SP **(D–F)** in the body **(A, D)**, trigone **(B, E)**, and fundus **(C, F)** after saline (control; *n* = 1) or RTX treatment (14 days post-application) at doses of 0.2 (*n* = 2), 1 (*n* = 2), or 2 (*n* = 2) μg/mL diluted with sterile saline to a total volume of 25 mL.

The treatment with RTX resulted in an approximate reduction of 8% (0.2 μg/mL, 318 nM), 55% (1 μg/mL, 1,591 nM) and 32% (2 μg/mL, 3,181 nM) of CGRP^+^ axons innervating the body of the urinary bladder (Figures 2A–D). Likewise, RTX treatment induced an approximate reduction of SP 24% (0.2 μg/mL, 318 nM), 63% (1 μg/mL, 1,591 nM), and 26% (2 μg/mL, 3,181 nM) of SP^+^ axons innervating the same anatomical region (Figures 2E–H). Quantitative analysis revealed that all intravesical RTX treatments reduced the density of both CGRP and SP axons in the body, trigone, and fundus of the urinary bladder (Figure 3).

**Figure 4:**
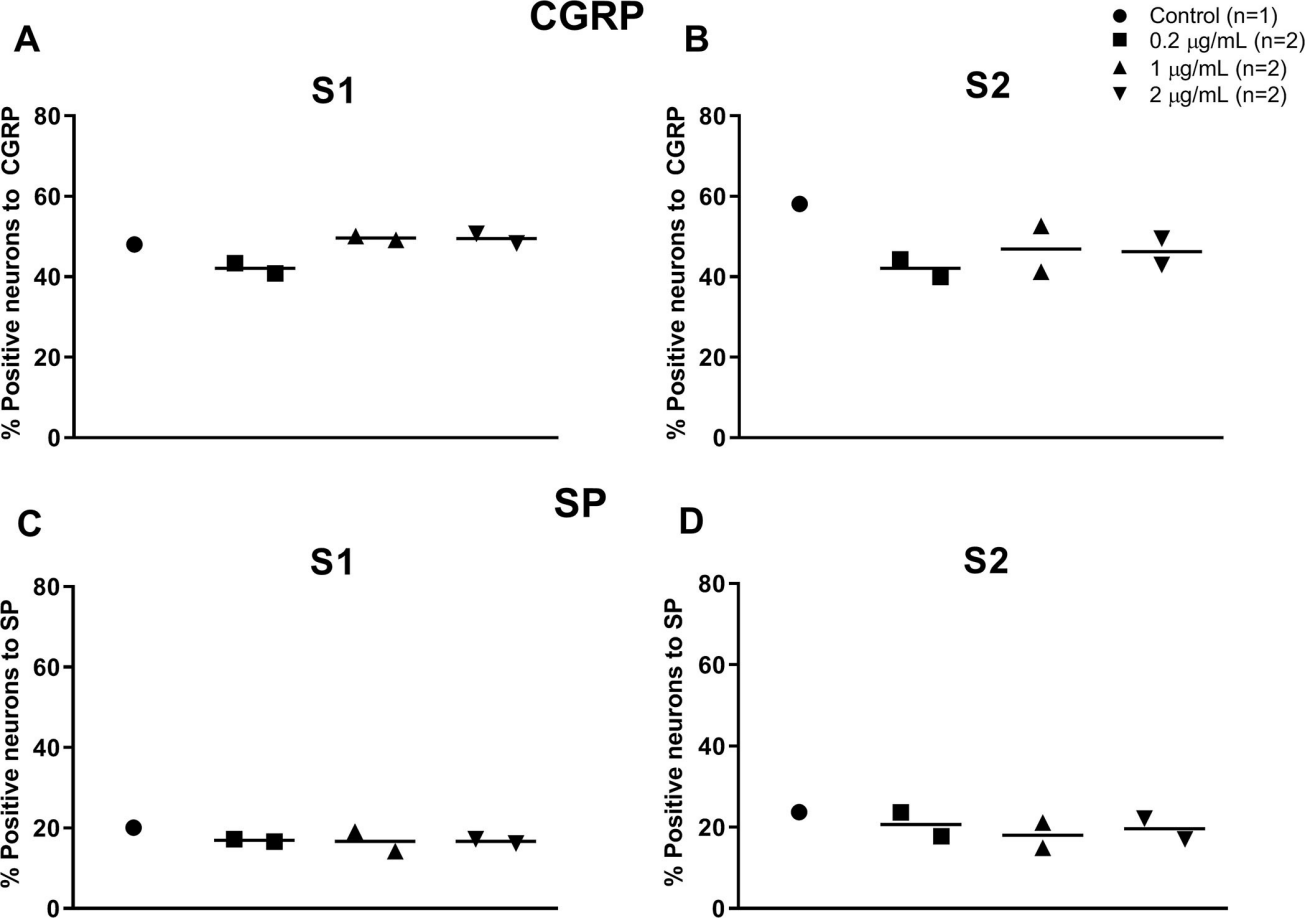
Individual values and median of cell bodies expressing CGRP **(A, B)** and SP **(C, D)** in S1 **(A, C)** and S2 **(B, D)** DRG of 7 healthy adult sexually intact female cats 14 days after instillation in the urinary bladder of saline (control; *n* = 1) or RTX treatment (14 days post-application) at doses of 0.2 (*n* = 2), 1 (*n* = 2), or 2 (*n* = 2) μg/mL diluted with sterile saline to a total volume of 25 mL.

In both S1 and S2 DRG samples, CGRP immunostaining was observed in the cell bodies and their associated axons. CGRP-positive cell bodies were small- to medium-size. Quantitative immunofluorescence analysis for SP in DRG revealed that this neuropeptide is expressed in fewer neurons compared to CGRP, and its expression is restricted to small- to medium-size primary afferent neurons. Quantitative analysis revealed that intravesical RTX treatment did not affect the percent of cell bodies expressing CGRP and SP at S1 and S2 DRG at any of the doses tested (Figure 4).

## Discussion

Application of RTX in the feline urinary bladder was well tolerated at all doses tested in this study. The only adverse effect noted was an increase in heart rate and non-detectable blood pressures in one subject that was maintained on a light plane of general anesthesia. A similar increase in heart rate, in conjunction with systemic hypertension, has been reported in dogs receiving RTX intrathecally. This seems to be caused by transient intense activation of the C-fibers lasting ~60 min (5, 26), which is the same time frame required by the cardiovascular variables of the subject in the present study to return to normal limits. However, in our study, the cat's blood pressure decreased before becoming undetectable. The hypotension could have been a result of decreased cardiac output due to the abrupt severe tachycardia. The instillation of 25 mL along with the activation of the C-fibers mediated by RTX could have led to a rapid increase in intravesical pressure. It is possible that this sudden and sustained change in pressure caused a vagally mediated decrease in cardiac output and the fall in blood pressure, similar to what has been described in a previous study in 26% of dogs after distension of the urinary bladder (27). Furthermore, the duration of the hypotensive state could have been lengthened by the vasodilatory effect of isoflurane. Thus, the concurrence of: (1) an increase in intravesical pressure, (2) the nociceptive response caused by the activation of the afferent pathways innervating the urinary bladder, and (3) the vasodilation produced by isoflurane could explain the occurrence and magnitude of the adverse event observed in this subject. The absence of any cardiovascular response in all the other cats that received additional ketamine indicates that the nociceptive activation may play a pivotal role in the adverse effects observed in the first subject. Due to this possible cardiovascular response observed in our study, it is advised to monitor the cardiovascular system during intravesical instillations of RTX while the subject is under general anesthesia. It is also recommended to ensure that the appropriate plane of anesthesia and analgesia is achieved before RTX application to decrease the occurrence and magnitude of potential cardiovascular events in response to the C-fiber activation. Another explanation to the abrupt decrease in blood pressure is an anaphylactic reaction to the treatment. Although this possibility cannot be excluded, it is unlikely due to the fact that it resolved without any specific treatment.

Data on systemic absorption of RTX after instillation in the urinary bladder are scarce. Only one study has been published where human patients with IC received a maximum concentration of 100 nM in 50 mL. Blood was collected at 15, 30, 45, and 60 min after administration of RTX and plasma concentrations were below the quantifiable limit at all time points (28). These findings are in agreement with our study, even if the cats received higher doses between 0.2 and 2 μg/mL (318–3,181 nM) in 25 mL. Plasma concentrations were above the quantifiable limits only in one subject at 30 min after treatment. This cat was the same that received 1 μg/mL (1,591 nM) of RTX and experienced abrupt tachycardia; however, the plasma levels of drug detected at 30 min were only 5.5 pg/mL (< 0.01 nM) above the quantifiable limits. Hence, a correlation between the systemic presence of RTX and the occurrence of the previously discussed cardiovascular changes seems unlikely.

Intravesical treatment with RTX resulted in dose-dependent changes in the epithelial tissue, lamina propria, and blood vessels in the urinary bladder. Similar results were found in rats exposed to 10 and 100 nM of RTX. The histological changes that could have been triggered by the transient activation of local C-fibers resolved at week 4 and 8 for the low and high dose, respectively (29). Another possible explanation is that the drug caused local irritation due to the activation of non-neuronal TRPV1 receptors such as those found in immune cells and epithelial cells of the urinary tract leading to the release of pro-inflammatory substances (30, 31). Based on the findings in rats (29), it is likely that the changes observed at the higher doses in our study are transient and self-limiting in nature. However, further studies with longer follow-up times would be needed to confirm this hypothesis.

RTX reduced the density of CGRP and SP immunoreactive axons in the feline urinary bladder. Despite the small number of cats per treatment group, the results suggest that significant ablation could be reached at dose of 1 μg/mL (1,591 nM). Similar findings were reported by Avelino et al., where 0.5 mL of 1, 10, 100, and 1,000 nM was instilled in the urinary bladder of rats. Maximal desensitization of the primary afferent fibers was reached at 100 nM (32). It is possible that a ceiling effect is reached at the aforementioned concentrations. In our study, however, RTX did not affect the number of neuron profiles expressing CGPR and SP in S1 and S2 DRG, where the cell bodies are located for the majority of sensory axons innervating the urinary bladder (22). This is indicative of selective chemoablation of the nociceptive nerve fibers in the bladder sparing the respective neuronal perikarya at a site remote from the RTX application. When SP- and CGRP-immunoreactive DRG cell cultures were exposed to RTX, a marked reduction to elimination of the neurites was observed in a dose-dependent manner; however, only a moderate decrease in cell bodies was noted (16). The authors concluded that sensory neurites were more sensitive to RTX than the soma, which could also explain why CGRP and SP in the DRG were not affected in the current study. Similar results were reported in rats, dogs, and a human subject where intrathecal injection of RTX resulted in ablation of TRPV1(+) central axonal projections and preservation of the neuronal somas (33).

Other findings in rats also suggest that local application of RTX only decreases the amount of SP and CGRP with complete pain desensitization without nerve degeneration (34). This could lead to recurrence of clinical signs after treatment with RTX due to neuroplasticity and prolonged or multiple applications may therefore be superior to a single treatment as was suggested for human patients (35).

Ablation of the TRPV1-expressing peripheral sensory nerve fibers is particularly advantageous in blocking inflammatory associated pain states. This mechanistic rationale supports the use of RTX as a therapeutic intervention in painful urinary bladder conditions, such as human and feline IC, which have many features in common (19, 36). Several studies on the use of RTX as a treatment for this syndrome have been published in people. When a single dose was used, an improvement of symptoms up to 3 months either statistically significant (37) or not (28) was noted in 2 studies. Another report showed decreased urinary bladder pain in 2 out of 4 patients (38). Results are even more promising when RTX is instilled in the urinary bladder *via* prolonged infusion over 10 days (39) or as a weekly treatment for 4 weeks (40). With the first dose regimen, the RTX group had a significantly lower pain score than the placebo, and in the second study 54% of patients reported good-to-excellent improvement of clinical signs. However, these results are in contrast with a large randomized, double-blind, placebo-controlled study conducted in 163 human patients with IC. People enrolled in the study were treated with 50 mL of RTX at 10, 50, 100 nM or placebo. After a follow-up period of 4 weeks, RTX failed to improve clinical signs (41).

Only one short communication investigated the effect of RTX in two cats with IC. These animals presented with upregulated vesicosympathetic cardiovascular and respiratory reflexes, which normalized after instillation of 100 nM of RTX for 30 min (42). At present, several treatments have been suggested for feline IC, including environmental enrichment, pheromones, nutritional management, administration of glycosaminoglycans and amitriptyline; however, the supportive scientific evidence is limited (43). RTX has the potential to reduce or eliminate clinical signs associated with feline IC and the current study revealed that 1 μg/mL (1,591 nM) could be used as starting dose for future clinical trials.

There are some limitations to the current study. We only enrolled a small number of animals. The goal was to determine the maximum dose tolerated that produced a decrease in SP and CGRP with minimal clinical adverse effects and histopathological changes. No previous studies have been published in cats and we did not want to sacrifice a larger number of animals without validating the protocol.

To confirm the presence of SP and CGRP in the feline urinary bladder and DRG and their response to RTX treatment, we only included healthy subjects. No data on the role of CGRP in feline IC have been published and only two reports studied SP in cats affected by this syndrome (20, 21). Although SP increases in inflammatory processes of the urinary bladder, it is still unclear whether the amount of these two neuropeptides is proportional to the severity of the disease and whether they can be used to monitor the response to the treatment and the resolution of clinical signs. It is possible that SP and CGRP will respond differently to RTX in cats with a pathologic urinary bladder.

For the immunofluorescence quantification of CGRP we used the anti-CGRP antibody by Sigma, since it has been validated by the company in feline tissues. The antibody in our study used the same immunogen that was validated in cat tissues by Gibson (25); however, we did not validate ourselves this antibody in the feline urinary bladder and DRG tissues.

Animals were observed for 14 days after treatment. This time was chosen to detect any potential reversible histological effects of RTX on the urinary bladder related to the initial activation of C-fibers. Changes were evident in the tissues examined; however, a longer follow-up time would be required to determine whether or not these changes are reversible. Although a 14-day period was sufficient to observe any acute adverse effects to the treatment, we cannot make any predictions on possible long-term complications.

The results of the present study suggest that a dose of 1 μg/mL (1,591 nM) of RTX in 25 mL of saline instilled for 20 min in the urinary bladder of healthy cats decreases SP and CGRP peripherally but not in S1 and S2 DRG. During the administration, a cardiovascular response can occur if the animal is under a light plane of anesthesia and not pretreated with an antinociceptive drug. A dose of 1 μg/mL (1,591 nM) also induces moderate changes in the urinary bladder tissue without causing any clinical signs. Future studies investigating the short and long-term effects of this treatment in cats with IC are warranted.

## Data availability statement

The raw data supporting the conclusions of this article will be made available by the authors, without undue reservation.

## Ethics statement

The animal study was reviewed and approved by the University of Georgia Institutional Animal Care and Use Committee.

## Author contributions

MB participated in study design, data collection and interpretation, manuscript preparation, and editing and reviewing. JG, AE, KM, HNT, and JAG participated in data collection and manuscript preparation. JJ-A participated in histologic analysis, data interpretation, and manuscript preparation. AN and AC participated in study design, manuscript preparation, and editing and reviewing. All authors contributed to the article and approved the submitted version.

## References

[1] Adolf W Sorg B Hergenhahn M Hecker E. Structure-activity relations of polyfunctional diterpenes of the daphnane type. I Revised structure for resiniferatoxin and structure-activity relations of resiniferonol and some of its esters. J Nat Prod. (1982) 45:347–54. 10.1021/np50021a018 7119808

[2] Szallasi A Blumberg PM. Resiniferatoxin, a phorbol-related diterpene, acts as an ultrapotent analog of capsaicin, the irritant constituent in red pepper. Neuroscience. (1989) 30:515–20. 10.1016/0306-4522(89)90269-8 2747924

[3] Olah Z Szabo T Karai L Hough C Fields RD Caudle RM. Ligand-induced dynamic membrane changes and cell deletion conferred by vanilloid receptor 1. J Biol Chem. (2001) 276:11021–30. 10.1074/jbc.M008392200 11124944

[4] Bates BD Mitchell K Keller JM Chan CC Swaim WD Yaskovich R. Prolonged analgesic response of cornea to topical resiniferatoxin, a potent TRPV1 agonist. Pain. (2010) 149:522–8. 10.1016/j.pain.2010.03.024 20403666 PMC2913152

[5] Brown DC Agnello K Iadarola MJ. Intrathecal resiniferatoxin in a dog model: efficacy in bone cancer pain. Pain. (2015) 156:1018–24. 10.1097/j.pain.0000000000000115 25659068 PMC4431903

[6] Karai L Brown DC Mannes AJ Connelly ST Brown J Gandal M. Deletion of vanilloid receptor 1-expressing primary afferent neurons for pain control. J Clin Invest. (2004) 113:1344–52. 10.1172/JCI20449 15124026 PMC398431

[7] Bishnoi M Bosgraaf CA Premkumar LS. Preservation of acute pain and efferent functions following intrathecal resiniferatoxin-induced analgesia in rats. J Pain. (2011) 12:991–1003. 10.1016/j.jpain.2011.03.005 21680254 PMC3645374

[8] Leo M Schulte M Schmitt LI Schafers M Kleinschnitz C Hagenacker T. Intrathecal resiniferatoxin modulates TRPV1 in DRG neurons and reduces TNF-induced pain-related behavior. Mediators Inflamm. (2017) 2017:2786427. 10.1155/2017/2786427 28831207 PMC5558708

[9] Raithel SJ Sapio MR LaPaglia DM Iadarola MJ Mannes AJ. Transcriptional changes in dorsal spinal cord persist after surgical incision despite preemptive analgesia with peripheral resiniferatoxin. Anesthesiology. (2018) 128:620–35. 10.1097/ALN.0000000000002006 29271803 PMC11175836

[10] Salas MM Clifford JL Hayden JR Iadarola MJ Averitt DL. Local resiniferatoxin induces long-lasting analgesia in a rat model of full thickness thermal injury. Pain Med. (2017) 18:2453–65. 10.1093/pm/pnw260 27794548 PMC6279302

[11] Kim Y Kim EH Lee KS Lee K Park SH Na SH. The effects of intra-articular resiniferatoxin on monosodium iodoacetate-induced osteoarthritic pain in rats. Korean J Physiol Pharmacol. (2016) 20:129–36. 10.4196/kjpp.2016.20.1.129 26807032 PMC4722186

[12] Iadarola MJ Sapio MR Raithel SJ Mannes AJ Brown DC. Long-term pain relief in canine osteoarthritis by a single intra-articular injection of resiniferatoxin, a potent TRPV1 agonist. Pain. (2018) 159:2105–14. 10.1097/j.pain.0000000000001314 30015705 PMC8121156

[13] Price TJ Flores CM. Critical evaluation of the colocalization between calcitonin gene-related peptide, substance P, transient receptor potential vanilloid subfamily type 1 immunoreactivities, and isolectin B4 binding in primary afferent neurons of the rat and mouse. J Pain. (2007) 8:263–72. 10.1016/j.jpain.2006.09.005 17113352 PMC1899162

[14] Buck SH Burks TF. The neuropharmacology of capsaicin: review of some recent observations. Pharmacol Rev. (1986) 38:179–226. 3534898

[15] Holzer P. Local effector functions of capsaicin-sensitive sensory nerve endings: involvement of tachykinins, calcitonin gene-related peptide and other neuropeptides. Neuroscience. (1988) 24:739–68. 10.1016/0306-4522(88)90064-4 3288903

[16] Jeftinija S Liu F Jeftinija K Urban L. Effect of capsaicin and resiniferatoxin on peptidergic neurons in cultured dorsal root ganglion. Regul Pept. (1992) 39:123–35. 10.1016/0167-0115(92)90534-2 1279751

[17] Hockman TM Cisternas AF Jones B Butt MT Osborn KG Steinauer JJ. Target engagement and histopathology of neuraxial resiniferatoxin in dog. Vet Anaesth Analg. (2018) 45:212–26. 10.1016/j.vaa.2017.10.005 29361418

[18] Sharrad DF Hibberd TJ Kyloh MA Brookes SJ Spencer NJ. Quantitative immunohistochemical co-localization of TRPV1 and CGRP in varicose axons of the murine oesophagus, stomach and colorectum. Neurosci Lett. (2015) 599:164–71. 10.1016/j.neulet.2015.05.020 25980991

[19] Buffington CA. Idiopathic cystitis in domestic cats–beyond the lower urinary tract. J Vet Intern Med. (2011) 25:784–96. 10.1111/j.1939-1676.2011.0732.x 21564297 PMC3760680

[20] Birder LA Wolf-Johnston AS Chib MK Buffington CA Roppolo JR Hanna-Mitchell AT. Beyond neurons: involvement of urothelial and glial cells in bladder function. Neurourol Urodyn. (2010) 29:88–96. 10.1002/nau.20747 20025015 PMC2910110

[21] Buffington CA Wolfe SA Jr. High affinity binding sites for [3H]substance P in urinary bladders of cats with interstitial cystitis. J Urol. (1998) 160:605–11. 10.1016/S0022-5347(01)62967-7 9679937

[22] Downie JW Champion JA Nance DM A. quantitative analysis of the afferent and extrinsic efferent innervation of specific regions of the bladder and urethra in the cat. Brain Res Bull. (1984) 12:735–40. 10.1016/0361-9230(84)90154-0 6206931

[23] Sculptoreanu A de Groat WC Buffington CA Birder LA. Abnormal excitability in capsaicin-responsive DRG neurons from cats with feline interstitial cystitis. Exp Neurol. (2005) 193:437–43. 10.1016/j.expneurol.2005.01.011 15869946

[24] Gregg TR Siegel A. Differential effects of NK1 receptors in the midbrain periaqueductal gray upon defensive rage and predatory attack in the cat. Brain Res. (2003) 994:55–66. 10.1016/j.brainres.2003.09.024 14642448

[25] Gibson SJ Polak JM Bloom SR Sabate IM Mulderry PM Ghatei MA. Calcitonin gene-related peptide immunoreactivity in the spinal cord of man and of eight other species. J Neurosci. (1984) 4:3101–11. 10.1523/JNEUROSCI.04-12-03101.1984 6209366 PMC6564846

[26] Brown DC Iadarola MJ Perkowski SZ Erin H Shofer F Laszlo KJ. Physiologic and antinociceptive effects of intrathecal resiniferatoxin in a canine bone cancer model. Anesthesiology. (2005) 103:1052–9. 10.1097/00000542-200511000-00020 16249680

[27] Nemeth CJ Khan RM Kirchner P Adams R. Changes in canine bladder perfusion with distension. Invest Urol. (1977) 15:149–50. 903211

[28] Chen TY Corcos J Camel M Ponsot Y Tu le M. Prospective, randomized, double-blind study of safety and tolerability of intravesical resiniferatoxin (RTX) in interstitial cystitis (IC). Int Urogynecol J Pelvic Floor Dysfunct. (2005) 16:293–7. 10.1007/s00192-005-1307-4 15818465

[29] Craft RM Cohen SM Porreca F. Long-lasting desensitization of bladder afferents following intravesical resiniferatoxin and capsaicin in the rat. Pain. (1995) 61:317–23. 10.1016/0304-3959(94)00193-I 7659443

[30] Avelino A Cruz F. TRPV1 (vanilloid receptor) in the urinary tract: expression, function and clinical applications. Naunyn Schmiedebergs Arch Pharmacol. (2006) 373:287–99. 10.1007/s00210-006-0073-2 16721555

[31] Khalil M Alliger K Weidinger C Yerinde C Wirtz S Becker C. Functional role of transient receptor potential channels in immune cells and epithelia. Front Immunol. (2018) 9:174. 10.3389/fimmu.2018.00174 29467763 PMC5808302

[32] Avelino A Cruz F Coimbra A. Intravesical resiniferatoxin desensitizes rat bladder sensory fibres without causing intense noxious excitation. A c-fos study. Eur J Pharmacol. (1999) 378:17–22. 10.1016/S0014-2999(99)00451-3 10478560

[33] Sapio MR Neubert JK LaPaglia DM Maric D Keller JM Raithel SJ. Pain control through selective chemo-axotomy of centrally projecting TRPV1+ sensory neurons. J Clin Invest. (2018) 128:1657–70. 10.1172/JCI94331 29408808 PMC5873867

[34] Avelino A Cruz F. Peptide immunoreactivity and ultrastructure of rat urinary bladder nerve fibers after topical desensitization by capsaicin or resiniferatoxin. Auton Neurosci. (2000) 86:37–46. 10.1016/S1566-0702(00)00204-6 11269923

[35] Mourtzoukou EG Iavazzo C Falagas ME. Resiniferatoxin in the treatment of interstitial cystitis: a systematic review. Int Urogynecol J Pelvic Floor Dysfunct. (2008) 19:1571–6. 10.1007/s00192-008-0663-2 18563284

[36] Buffington CA Chew DJ DiBartola SP. Interstitial cystitis in cats. Vet Clin North Am Small Anim Pract. (1996) 26:317–26. 10.1016/S0195-5616(96)50212-3 8711867

[37] Lazzeri M Beneforti P Spinelli M Zanollo A Barbagli G Turini D. Intravesical resiniferatoxin for the treatment of hypersensitive disorder: a randomized placebo controlled study. J Urol. (2000) 164:676–9. 10.1097/00005392-200009010-00014 10953124

[38] Apostolidis A Gonzales GE Fowler CJ. Effect of intravesical Resiniferatoxin (RTX) on lower urinary tract symptoms, urodynamic parameters, and quality of life of patients with urodynamic increased bladder sensation. Eur Urol. (2006) 50:1299–305. 10.1016/j.eururo.2006.04.006 16697519

[39] Lazzeri M Spinelli M Beneforti P Malaguti S Giardiello G Turini D. Intravesical infusion of resiniferatoxin by a temporary *in situ* drug delivery system to treat interstitial cystitis: a pilot study. Eur Urol. (2004) 45:98–102. 10.1016/S0302-2838(03)00418-4 14667524

[40] Peng CH Kuo HC. Multiple intravesical instillations of low-dose resiniferatoxin in the treatment of refractory interstitial cystitis. Urol Int. (2007) 78:78–81. 10.1159/000096940 17192738

[41] Payne CK Mosbaugh PG Forrest JB Evans RJ Whitmore KE Antoci JP. Intravesical resiniferatoxin for the treatment of interstitial cystitis: a randomized, double-blind, placebo controlled trial. J Urol. (2005) 173:1590–4. 10.1097/01.ju.0000154631.92150.ef 15821499

[42] March P Teng B Westropp J Buffington T. Effects of resiniferatoxin on the neurogenic component of feline interstitial cystitis. Urology. (2001) 57:114. 10.1016/S0090-4295(01)01054-8 11378087

[43] Forrester SD Towell TL. Feline idiopathic cystitis. Vet Clin North Am Small Anim Pract. (2015) 45:783–806. 10.1016/j.cvsm.2015.02.007 25813400

